# Evaluation on the Nanoscale Zero Valent Iron Based Microbial Denitrification for Nitrate Removal from Groundwater

**DOI:** 10.1038/srep12331

**Published:** 2015-07-22

**Authors:** Lai Peng, Yiwen Liu, Shu-Hong Gao, Xueming Chen, Pei Xin, Xiaohu Dai, Bing-Jie Ni

**Affiliations:** 1 Advanced Water Management Centre, The University of Queensland, St Lucia, Brisbane, Queensland 4072, Australia; 2 State Key Laboratory of Hydrology-Water Resources and Hydraulic Engineering, Hohai University, Nanjing, China; 3 State Key Laboratory of Pollution Control and Resources Reuse, National Engineering Research Center for Urban Pollution Control, College of Environmental Science and Engineering, Tongji University, Shanghai 200092, PR China

## Abstract

Nanoscale zero valent iron (NZVI) based microbial denitrification has been demonstrated to be a promising technology for nitrate removal from groundwater. In this work, a mathematical model is developed to evaluate the performance of this new technology and to provide insights into the chemical and microbial interactions in the system in terms of nitrate reduction, ammonium accumulation and hydrogen turnover. The developed model integrates NZVI-based abiotic reduction of nitrate, NZVI corrosion for hydrogen production and hydrogen-based microbial denitrification and satisfactorily describes all of the nitrate and ammonium dynamics from two systems with highly different conditions. The high NZVI corrosion rate revealed by the model indicates the high reaction rate of NZVI with water due to their large specific surface area and high surface reactivity, leading to an effective microbial nitrate reduction by utilizing the produced hydrogen. The simulation results further suggest a NZVI dosing strategy (3–6 mmol/L in temperature range of 30–40 °C, 6–10 mmol/L in temperature range of 15–30 °C and 10–14 mmol/L in temperature range of 5–15 °C) during groundwater remediation to make sure a low ammonium yield and a high nitrogen removal efficiency.

Nitrate (NO_3_^−^) has been recognized as the most ubiquitous chemical contaminant in aquifers^1^. The level of nitrate in groundwater has been increasing as a result of the use of nitrogen-enriched fertilizers and irrigation with domestic wastewater^2^. Nitrate contamination in surface water and ground water has received increasing attentions owing to its potential to cause health and eutrophication problems^3^. The possible health consequences of nitrate ingestion involves methemoglobinemia in infants after nitrate transformation into nitrite and the formation of carcinogenic nitrosamines after reacting with secondary or tertiary amines^2^. According to the US Environmental Protection Agency’s standard, the acute toxicity of nitrate generally has been set at concentrations greater than 50 mg/L and the maximum contaminant level been documented at 10 mg/L^1^.

Traditional treatment process for nitrate removal includes ion exchange, reverse osmosis and electrodialysis, which are expensive in operation and disposal of the produced concentrated waste^2^. Alternative nitrate removal approaches involves physical adsorption, chemical reduction and biological denitrification. Physical adsorption using adsorbent materials are not proper for *in-situ* applications due to the presence of impurities in aquifers, while chemical reduction, in particular the zero valent iron (ZVI) has been widely applied for groundwater remediation and wastewater treatment due to low expense and its ability to reduce oxidized pollutants^2^,^4^,^5^,^6^,^7^,^8^,^9^. In comparison to the granular ZVI, nanoscale zero-valent iron (NZVI) possesses larger specific surface area and higher surface reactivity and would be more efficient in treating contaminants in groundwater and wastewater^5^,^10^. Nitrate could be reduced chemically by NZVI^11^. However, ammonium (NH_4_^+^) as the end product of this abiotic reaction would cause eutrophication problem and need to be further treated by microbial attenuation, which involves combined nitrification and denitrification process to convert ammonium to nitrogen gas^12^.

Biological denitrification serves to be an alternative option for nitrate removal, which has been extensively applied in wastewater treatment. However, heterotrophic denitrification would produce excessive biomass and soluble microbial products that require subsequent treatment prior to water utilization^13^. In contrast, nitrate could be removed more cleanly with minimal biomass yield by using autotrophic hydrogen-oxidizing denitrifiers^14^,^15^,^16^. Since the use of hydrogen gas (H_2_) in engineered denitrification system is quite challenging taking into account the high cost and the explosive properties, hydrogen gas generation by anaerobic iron corrosion may overcome the limitations associated with hydrogen-utilizing denitrification^17^,^18^,^19^. The NZVI-based microbial hydrogen-utilizing denitrification for nitrate removal has been demonstrated to be a promising approach^13^,^20^. The NZVI dosage and temperature have a significant influence on nitrate removal in such systems.

In this work, a mathematical model describing the NZVI-based microbial hydrogen-utilizing denitrification is developed to evaluate the system performance and to provide insights into the chemical and microbial interactions involved in the system. Both the abiotic (chemical nitrate reduction and NZVI corrosion) and biological processes (microbial nitrate reduction) that would occur in such systems are taken into account. The model is calibrated and validated with independent experimental data sets obtained from two different systems under different conditions. Model-based optimization is also performed in terms of the key application conditions, i.e., NZVI dosing concentration and application temperature.

## Results

### Evaluation on NZVI-based microbial nitrate reduction in System I

**Figure 1:**
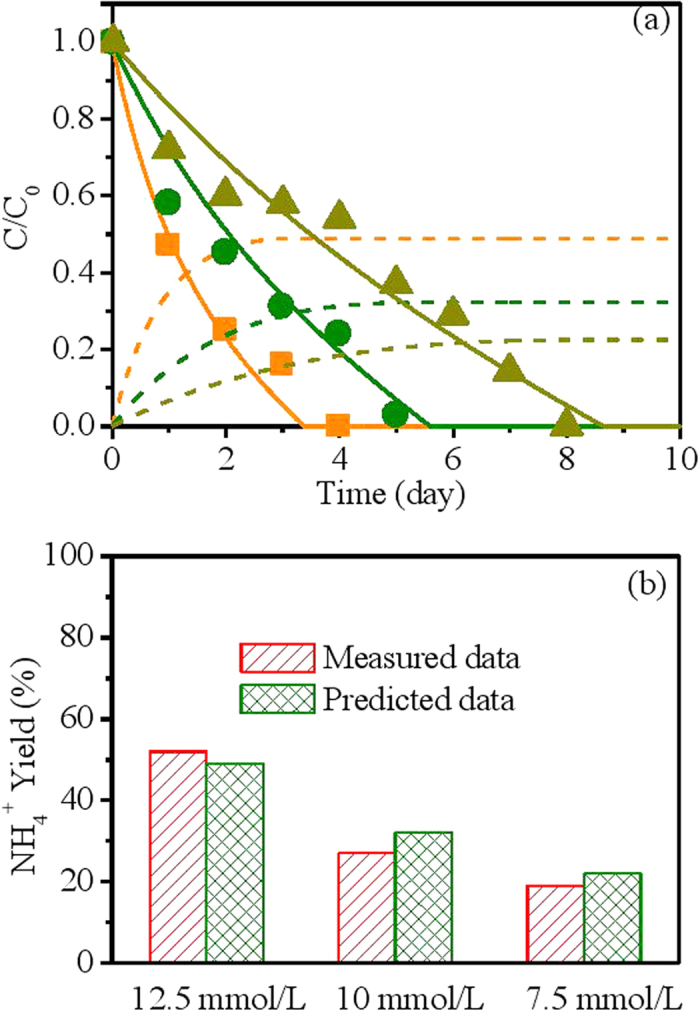
Model calibration results using batch data with varying NZVI dosage from System I. (Figure 1a: 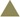, measured nitrate data at NZVI concentration of 7.5 mmol/L; 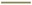, predicted nitrate data at NZVI concentration of 7.5 mmol/L; 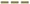, predicted ammonium data at NZVI concentration of 7.5 mmol/L; 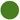, measured nitrate data at NZVI concentration of 10 mmol/L; 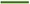, predicted nitrate data at NZVI concentration of 10 mmol/L; 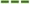, predicted ammonium data at NZVI concentration of 10 mmol/L; 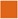, measured nitrate data at NZVI concentration of 12.5 mmol/L; 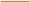, predicted nitrate data at NZVI concentration of 12.5 mmol/L; 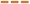, predicted ammonium data at NZVI concentration of 12.5 mmol/L).

The calibration of the developed model involved optimizing key parameter values for nitrate reduction by fitting simulation results to batch test data. The experimentally observed and model predicted nitrate data (the ratio between measured nitrate concentration and initial nitrate concentration) and ammonium yield (the ratio between measured ammonium concentration and initial nitrate concentration) at varying NZVI dosing concentrations are shown in Fig. 1. At all NZVI concentrations (7.5 mmol/L, 10 mmol/L and 12.5 mmol/L), the nitrate in the combined system based on measurement was completely removed within 9 days. Both of the nitrate removal rate (Fig. 1a) and ammonium yield (Fig. 1b) increased with the increase of NZVI dosage. The good agreement between these simulated and measured data at varying level of NZVI dosing concentrations indicates that the developed model properly predicts the effect of NZVI dosage on nitrate removal and ammonium yield in System I (Fig. 1).

**Figure 2:**
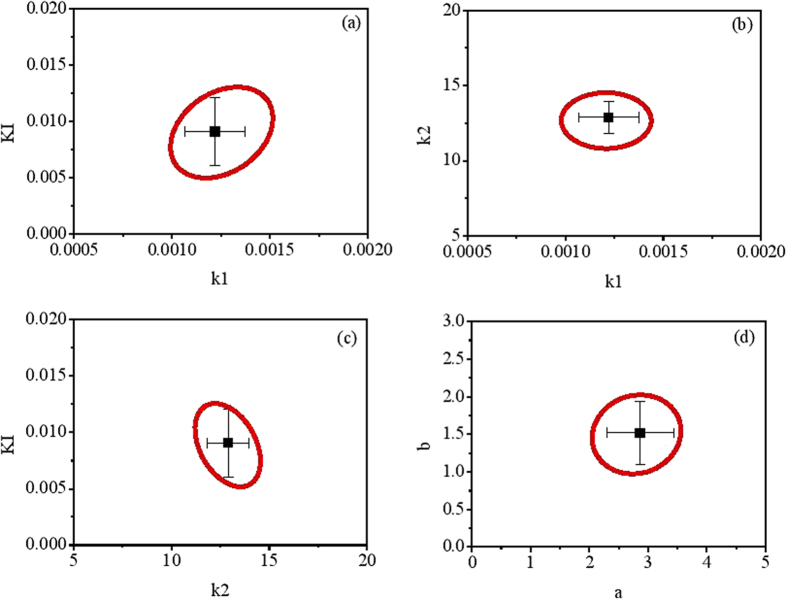
95% confidence regions for the parameter combinations among the key model parameters with the best fits in the center, as well as their standard errors: (**a**) k_1_ vs. K_I_; (**b**) k_1_ vs. k_2_; (**c**) k_2_ vs. K_I_; (**d**) a vs. b.

The calibrated parameter values giving the optimum fit are listed in Table 1. Parameter uncertainty analysis of a model structure is important as it informs which parameter combinations can be estimated with the given measured data. The parameter correlation matrix obtained from model calibration indicates most of the parameter combinations have not shown significant correlation, except for four of them with correlation coefficients greater than 0.8. Thus, these four parameter combinations were further analyzed to evaluate the uncertainty associated with their estimates. The two-parameter 95% confidence surfaces of the objective function for the degrees of correlation between the selected two parameters are shown in Fig. 2. The 95% confidence regions for all the four parameter pairs are bound by small ellipsoids having mean values for the parameter estimates approximately at the center, indicating good identifiability of these estimated parameters (Fig. 2). The 95% confidence intervals for all the single parameters are also small, which are generally within 10% of the estimated values (Fig. 2). These results indicate that these parameters have a high-level of identifiability and the estimated values are reliable.

**Figure 3:**
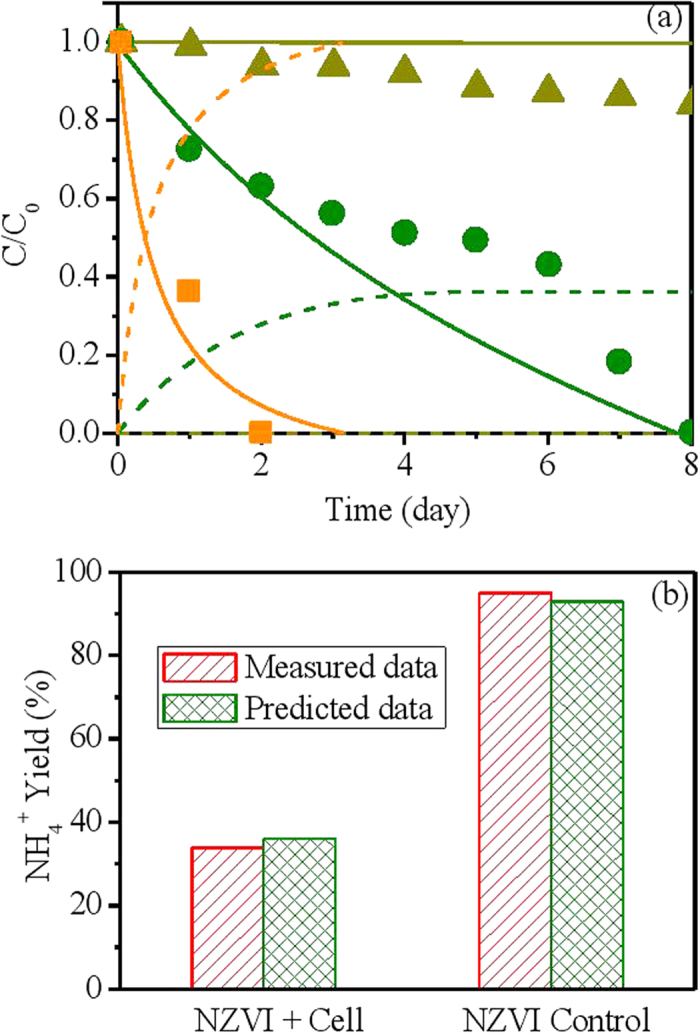
Model validation results using batch data of experimental group and control groups from System I. (Figure 3a: 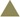, measured nitrate data in cell control group; 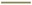, predicted nitrate data in cell control group; 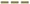, predicted ammonium data in cell control group; 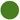, measured nitrate data in NZVI + cell group; 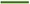, predicted nitrate data in NZVI + cell group; 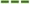, predicted ammonium data in NZVI + cell group; 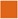, measured nitrate data in NZVI control group; 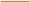, predicted nitrate data in NZVI control group; 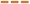, predicted ammonium data in NZVI control group).

Model validation aims to further test the validity and reliability of the estimated parameters obtained from model calibration (Table 1) by comparing the model predictions with these parameters to the experimental data from other batch tests in System I (not used for model calibration). The model was evaluated with three batch tests, namely NZVI control group (without biomass), cell control group (without NZVI) and NZVI + cell group (containing both NZVI and biomass). The experiment results and model predictions are shown in Fig. 3. Nitrate in cell control decreased slowly possibly due to the presence of small amount of fermentable organic materials. Complete nitrate removal was observed within 8 days with about 33% ammonium yield in NZVI + cell group, while all added nitrate was reduced within 2 days with over 95% ammonium yield in NZVI control group. Even though there are some discrepancies between model predictions and measurements in cell control group and NZVI + cell group (Fig. 3a), likely due to the fact that the minor fermentation process is not considered in the model structure for simplicity and the available data for model calibration is relatively limiting, the developed model can generally capture the overall trend of nitrate data and produced ammonium data in all three groups, as well as the inhibitory effect of biomass on abiotic nitrate reduction (Fig. 3).

### Evaluation on NZVI-based microbial nitrate reduction in System II

The experimental results obtained from System II were also used to evaluate the developed model. The batch test data at different temperatures were used to calibrate the model. The obtained parameter values for System II shows some variations from those in System I due to the highly different operational conditions in these two cases (e.g. culture types, biomass concentration, NZVI dosage and temperature).

**Figure 4:**
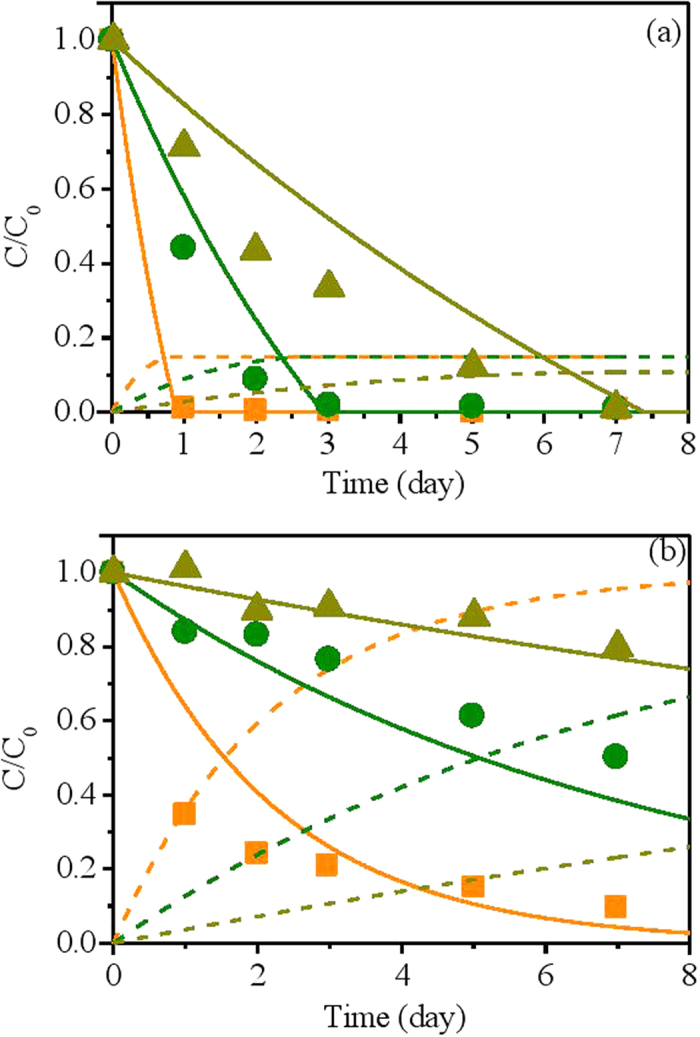
Model calibration results using (**a**) nitrate removal data in the combined system and (**b**) abiotic reduction of nitrate by NZVI at different temperatures from System II. (for both Fig. 3a,b: 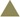, measured nitrate data at 12 °C; 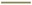, predicted nitrate data at 12 °C; 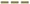, predicted ammonium data at 12 °C; 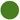, measured nitrate data at 25 °C; 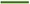, predicted nitrate data at 25 °C; 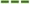, predicted ammonium data at 25 °C; 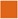, measured nitrate data at 37 °C; 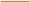, predicted nitrate data at 37 °C; 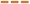, predicted ammonium data at 37 °C).

Model calibration results using i) nitrate removal data in the combined system and ii) abiotic reduction of nitrate by NZVI at different temperatures from System II are shown in Fig. 4. The nitrate removal rate for both systems increased as the temperature in the reactors increased from 12 °C to 37 °C. The predictions of the developed model were in good agreement with measured data at varying level of temperature in both systems.

**Figure 5:**
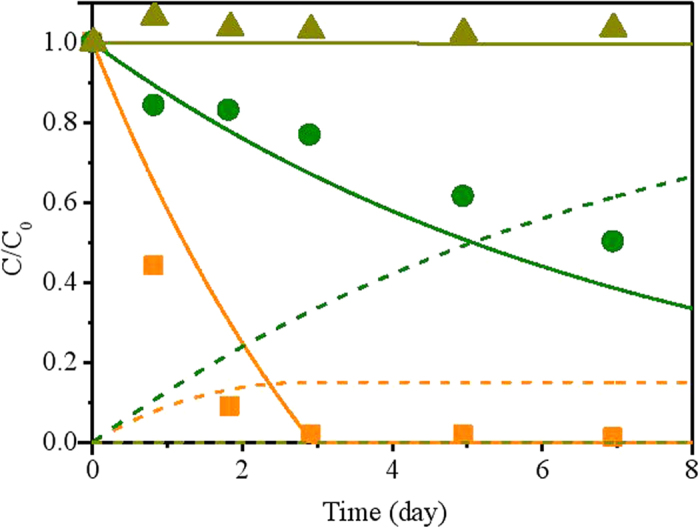
Model validation results using batch data of experimental group and control groups from System II. (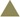, measured nitrate data in cell control group; 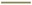, predicted nitrate data in cell control group; 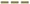, predicted ammonium data in cell control group; 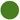, measured nitrate data in NZVI control group; 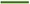, predicted nitrate data in NZVI control group; 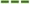, predicted ammonium data in NZVI control group; 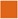, measured nitrate data in NZVI + cell group; 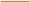, predicted nitrate data in NZVI + cell group; 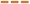, predicted ammonium data in NZVI + cell group).

Experimental data from NZVI control group (without biomass), cell control group (without NZVI) and NZVI + cell group (containing both NZVI and biomass) were further applied to validate the model (Fig. 5). The model could properly describe all of the trends of nitrate removal in the three groups, indicating a good validity of the developed model.

## Discussion

Increasing evidence shows that nitrate contaminated surface water and groundwater could be treated with a combination of NZVI and hydrogen-utilizing bacteria^13^,^20^. However, this treatment is limited by ammonium yield during abiotic nitrate reduction by NZVI, which would cause eutrophication problem. In this work a mathematical model that integrates abiotic reduction by NZVI, NZVI corrosion and hydrogen-based autotrophic denitrification has been developed to evaluate the performance of this new technology for the first time. The model calibration and validation using experimental data from two previously reported data demonstrated the proposed modelling approach could be applicable to systems under varying operational conditions.

Kinetic models for denitrification of hydrogen-utilizing bacteria has previously been developed based on different cultures^24^,^25^,^26^. In this work, the kinetics model of hydrogen-utilizing denitrification was coupled to two chemical reactions, namely nitrate reduction by NZVI and NZVI corrosion. The process of ZVI corrosion has previously been described by Xiao *et al*.^22^ using the Michaelis-Menten equation. Hence we adapted this equation directly. The calibrated value of maximum NZVI corrosion rate (k_2_ in Table 1) was two orders of magnitude higher than the reported value for ZVI corrosion rate in Xiao *et al*.^22^. The high NZVI corrosion rate revealed by the model indicates the high reaction rate of NZVI with water for hydrogen production due to their large specific surface area and high surface reactivity, leading to an effective microbial nitrate reduction by utilizing the produced hydrogen. The process of abiotic nitrate reduction by NZVI has not been described before. A pseudo multi-order kinetics was successfully applied in the new model to simulate this chemical reaction with the corresponding parameters (k_1_, a, b, and K_I_) newly obtained. It should be noted that the process of Fe (0) corrosion is the rate-limiting step for autotrophic denitrification process, due to the reaction rate of Fe (0) corrosion (Process 2 in Table 2) is much lower than that of autotrophic denitrification (Process 3 in Table 2). Hence, the variation of NZVI dosages would affect the steady-state biomass concentration indirectly via regulating the availability of electron donor (H_2_).

In System I and System II, the nitrate reduction was mainly attributed to two pathways including NZVI-based abiotic nitrate conversion to ammonium and hydrogen-based autotrophic denitrification that converts nitrate to N_2_. The relative contribution of each pathway to nitrate reduction could be identified based on the ammonium yield, which can only be generated by abiotic nitrate reduction by NZVI. The small amount of nitrate reduction owing to the presence of fermentable organic material was not considered in this model. Based on the measured data in System I (Fig. 3), the chemical reduction of nitrate in the NZVI control group (without biomass) is much faster than the nitrate reduction in the group containing both of NZVI and microbes, indicating that the biomass exerts a inhibitory effect on the chemical reaction between NZVI and nitrate possibly by resisting the electron transfer from NZVI to nitrate^20^. The increase of NZVI dosage from 7.5 mmol/L to 12.5 mmol/L increased the contribution of chemical nitrate reduction pathway from approximately 20% to 50%, accompanied by the decrease of contribution of biological nitrate reduction pathway (Fig. 1). In our model, the relative contributions of these two pathways are regulated by a biomass inhibitory term (K_I_/(K_I_ + X_AD_)) and the reaction rates of the abiotic nitrate reduction and NZVI corrosion. Our model managed to capture the shift of pathways in such a system (Figs 1 & 3).

However, the contributions of the chemical and biological pathways to nitrate reduction are completely different in System II. The biomass in System II is around 1000 mg VSS/L, which would exert a more obvious inhibitory effect on the chemical pathway than Case I (averaged 70 mg VSS/L). Our model predicted that in System II, the contribution of chemical nitrate reduction pathway only accounts for no more than 15% (Figs 4 & 5). The simulation results also indicated that the elevated temperature increased the nitrate reduction from both pathways, but would not change the relative contributions a lot (Figs 4 & 5).

**Figure 6:**
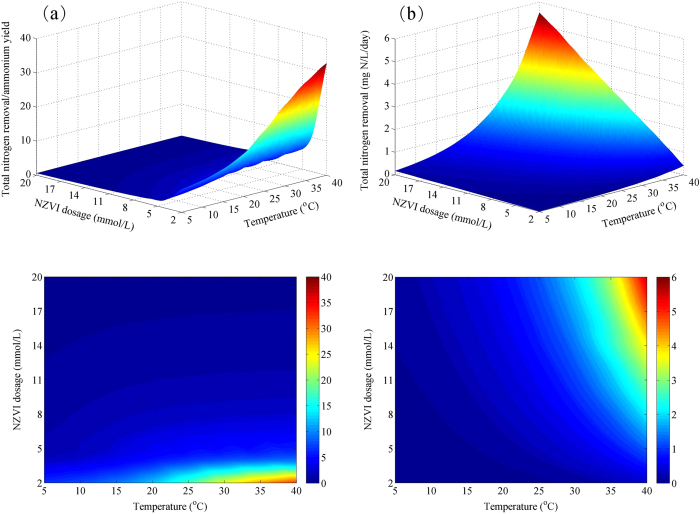
Model simulation results of (**a**) total nitrogen removal/ammonium yield and (**b**) total nitrogen removal at varying temperatures and NZVI dosing concentrations.

Groundwater temperature is found to strongly linked to air temperature, ranging from 5 to 25 °C across the USA, with potential higher (>25 °C) groundwater temperature in tropical areas with high annual air temperature^27^. In this study, the effect of NZVI dosage on nitrate reduction and ammonium yield is investigated under varying temperatures ranged from 5 to 40 °C, in order to cover a broad range of site-specific and seasonally dynamic groundwater temperature. We perform simulations with variations of temperature and NZVI dosage using the developed model in this study. Total nitrogen removal/ammonium yield (TNR/AY) and total nitrogen removal (TNR) were used as two indicators in the model simulation. Figure 6a,b illustrate the TNR/AY and TNR of the system with the combination of NZVI and hydrogen utilizing denitrification under varying conditions of NZVI dosage and temperature. At each NZVI dosage level, both of simulated TNR/AY and TNR increased rapidly as temperature increases from 5 to 40 °C. At each temperature level, with the increase of NZVI dosage from 2 to 20 mmol/L, the simulated TNR/AY decreased dramatically, while the simulated TNR increased rapidly. Figure 6 and Fig. 4a suggests that high temperature substantially increased the removal efficiency of total nitrogen, but has little effect on ammonium yield. The simulation results in Fig. 6 further indicate that high levels of NZVI dosage would increase nitrogen removal efficiency, but result in substantial ammonium yield, while low levels of NZVI dosage would affect the removal efficiency, but yield negligible ammonium. The region for high-level TNR/AY (>15) is limited to NZVI dosage of 2–3 mmol/L and temperature of 25–40 °C, whilst the region for high-level TNR (>2 mg N/L/day) is limited to NZVI dosage of 8–20 mmol/L and temperature of 30–40 °C. There is no overlapped region for maximum nitrate removal and minimum ammonium yield. Hence, a wise choice of NZVI dosage according to the temperature in groundwater is essential to make sure a relatively high nitrogen removal efficiency and a relatively low ammonium generation. Therefore, it is suggested that (1) in temperature range of 30–40 °C, NZVI dosage of 3–6 mmol/L should be applied; (2) in temperature range of 5–15 °C, NZVI dosage of 10–14 mmol/L should be applied; (3) within temperature range of 15–30 °C, NZVI dosage between 6–10 mmol/L should be used.

In summary, a new mathematical model that integrates abiotic nitrate reduction by NZVI, NZVI corrosion and hydrogen-based autotrophic denitrification was developed in this study. This mathematical model has been applied successfully to reproduce data of nitrate reduction and ammonium yield obtained from two systems with highly different operational conditions. The effects of NZVI dosage and temperature on nitrate reduction and ammonium yield could be fully described by this model. The simulations using this developed model suggest a strategy of NZVI dosages according to varying temperatures (3–6 mmol/L in temperature range of 30–40 °C, 6–10 mmol/L in temperature range of 15–30 °C and 10–14 mmol/L in temperature range of 5–15 °C) during groundwater remediation to make sure a low ammonium yield and a high efficiency of total nitrogen removal.

## Methods

### Model development

The new mathematical model synthesizes all relevant reactions involved in consumption and production of NO_3_^−^, NH_4_^+^, N_2_, NZVI (Fe (0)), ferrous ion (Fe (II)) and H_2_ in the NZVI-based system with microbial hydrogen-utilizing denitrification as below:

*Reaction 1: Abiotic nitrate reduction by NZVI*


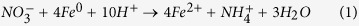


In this reaction, the NO_3_^−^ is reduced chemically by NZVI, whilst NH_4_^+^ and ferrous ion are generated simultaneously.

*Reaction 2: NZVI corrosion*


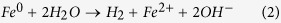


During NZVI corrosion, NZVI is converted into ferrous ion with H_2_ as one of the end products. The corrosion of NZVI is a significant process in the NZVI-based system as it provides electron donor (H_2_) for subsequent biological denitrification.

*Reaction 3: Hydrogen-utilizing denitrification*


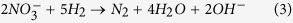


The hydrogen that originated from chemical NZVI corrosion can be utilized as the electron donor by autotrophic denitrifiers and proceeds to reduce nitrate to N_2_. This is the sole step for total nitrogen removal.

Based on above chemical and microbial reactions, the kinetics and stoichiometry of the developed model are summarized in Table 2, along with processes of microbial endogenous respiration and microbial inactivation of hydrogen-utilizing denitrifiers. The definitions, values, and units of all parameters used in the developed model are shown in Table 1. Kinetic control of all the enzymatic reaction rates is described by the Michaelis−Menten equation. The rate of microbial reaction is modelled by an explicit function of the concentrations of all substrates involved in the reaction, as described in detail in Table 2. The kinetics for NZVI corrosion is also described by the Michaelis-Menten equation^22^. A pseudo multiple order kinetics is used to simulate the abiotic nitrate reduction by NZVI (details refer to Table 2). The stoichiometry in Table 2 is determined based on mass and electron balance. For example, 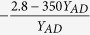 of nitrate stoichiometry in Table 2 is calculated based on nitrogen balance with N_2_ and electron balance with H_2_, N_2_ and X_AD_ according to COD-based units.

### Experimental data for model evaluation

Experimental data of nitrate removal at varying NZVI dosing concentrations and temperatures obtained from two different systems with combination of NZVI and hydrogen-utilizing denitrification are used for model calibration and validation^13^,^20^. The operational conditions of each case are briefly summarized as below:

*System I:*^20^ investigated the combination of NZVI particles with *Alcaligenes eutrophus* to remove nitrate under batch conditions. 10 mL stock solution with nitrate concentration of 50 mg N /L and 25 mL of the seed culture were added into a 175-mL serum bottle and was diluted to 100 mL with deionized water. The mixed liquor was then purged with Ar gas in order to remove residual oxygen and transferred into another serum bottle containing 0.056 g of NZVI particles. The bottle was sealed and mixed at 150 rpm using a rotary shaker at 30 °C after the initial pH was adjusted to 7.0 using HCl.

Batch experiment A compared nitrate reduction and ammonium production among the groups of cell control (without NZVI), NZVI control (without biomass) and NZVI + cell (containing both NZVI and biomass). Batch experiment B investigated the effect of three different initial contents of NZVI particles (7.5 mmol/L, 10 mmol/L and 12.5 mmol/L) on nitrate reduction and ammonium generation in the system with combination of NZVI and hydrogen-utilizing bacteria.

*System II:*^13^ studied the feasibility of applying NZVI to improve the microbial reduction of nitrate under batch conditions. The activated sludge from a wastewater treatment plant was used as the seed culture for the enrichment of microbial nitrate reduction culture. Batch experiments were carried out in a 250-ml amber bottles containing 100 mL medium and 0.5 g NZVI under anoxic and light-excluded conditions. The mixed liquor in amber bottles was purged with N_2_ for 10 min to remove any residual dissolved oxygen and then completely sealed to maintain anaerobic conditions. The nitrate concentration in each reactor was 10 mM. All bottles were placed on a platform shaker and continuously shaken during experiments.

Batch experiment A focused on evaluating nitrate reduction and ammonium production among the groups of cell control (without NZVI), NZVI control (without biomass) and NZVI + cell (containing both NZVI and biomass). Batch experiment B investigated the effect of different temperatures (12, 25 and 37 °C) on nitrate reduction and ammonium generation in the system with combination of NZVI and hydrogen-utilizing bacteria.

### Model calibration, uncertainty analysis and model validation

The developed model contains fourteen kinetic parameters, as summarized in Table 1. Eight of these parameters are well established in previous studies. Thus, literature values are directly adopted for these parameters (Table 1). For System I, the remaining five parameters are calibrated based on the experimental data from the batch experiment B in System I (Table 1). A inhibition term K_I_/(K_I_ + X_AD_) is applied to describe abiotic nitrate reduction, since System I clearly demonstrated an inhibitory effect of biomass on process 1 in Table 2. The parameters for the developed model in Table 1 was estimated by minimizing the sum of squares of the deviation between model predictions and experimental data from batch test B in system I, which is statistically reliable and has been widely applied for parameter estimation in water and wastewater treatment. AQUASIM was used to perform the estimation of parameters^28^. The objective function to be minimized in the parameter estimation is as follows^29^:


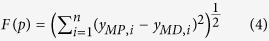


where y_MP,i_ and y_MD,i_ are model predictions and experimental data, respectively, at time t_i_ (i from 1 to n).

The experimental data from batch experiment A in System I are then used to validate the model with the calibrated model parameters.

Parameter estimation and parameter uncertainty evaluation are performed according to Batstone *et al*.^30^ with a 95% confidence level for significance testing and parameter uncertainty analysis. The standard errors and 95% confidence intervals of individual parameter estimates are calculated from the mean square fitting errors and the sensitivity of the model to the parameters. The determined F-values are used for parameter combinations and degrees of freedom in all cases. A modified version of AQUASIM 2.1d is used to determine the parameter surfaces^31^.

To further verify the validity and applicability of the model, we also applied the model to evaluate the experimental data from System II (details refer to Table 2). It should be noted that a temperature-dependent kinetics (k = k(25 °C)e^(c(T−25 °C))^) as described in Henze *et al*.^32^ was applied to process 1–3 of Table 2 to describe the effect of temperature on nitrate removal in System II (k is the maximum reaction rate at temperature of T °C; k(25 °C) is the maximum reaction rate at temperature of 25 °C, c is a constant for calibration).

### Model-based investigation on system performance at varying conditions

Since NZVI dosage and temperature are two key factors influencing the removal of nitrate contamination in groundwater^13^,^20^, both of them are investigated in the model simulations. The total nitrogen removal (TNR, mg N/L/day) and total nitrogen removal/ammonium yield (TNR/AY) are selected to represent the efficiency of contaminant removal in groundwater. Simulations of the developed model with calibrated data in Table 1 were conducted under eight temperature levels (5, 10, 15, 20, 25, 30, 35 and 40 °C) and ten NZVI dosing levels (2, 4, 6, 8, 10, 12, 14, 16,18, 20 mmol/L), which generated eighty sets of TNR as well as TNR/AY. The investigated levels of temperature and NZVI dosage cover a broad range of conditions in groundwater remediation.

## Additional Information

**How to cite this article**: Peng, L. *et al*. Evaluation on the Nanoscale Zero Valent Iron Based Microbial Denitrification for Nitrate Removal from Groundwater. *Sci. Rep*. **5**, 12331; doi: 10.1038/srep12331 (2015).

## Figures and Tables

**Table 1:** Kinetic and stoichiometric parameters of the developed model.

Parameter	Definition	System I	System II	Unit	Source
*Y*_*AD*_	yield coefficient for growth on S_H2_	0.00137	0.00137	kg COD g^−1^ H_2_	(1)
k_1_	Reaction rate on abiotic nitrate reduction	0.0015	0.137	d^−1^	estimated
k_2_	Reaction rate on Fe(0) corrosion	12.7	120	d^−1^	estimated
k_3_	Anoxic growth rate on NO_3_^−^ and H_2_	1.28	1.28	d^−1^	(1)
p_AD_	Endogenous respiration rate for AD	0.05	0.05	d^−1^	(1)
*b*_*AD*_	Inactivation coefficient of AD	0.05	0.05	d^−1^	(1)
*K*_*Fe(0)*_	*S*_*Fe(0)*_ affinity constant for H_2_ production	911	911	mol m^−3^	(2)
*K*_*NO3,AD*_	*S*_*NO3*_ affinity constant for nitrate reduction	0.18	0.18	g N m^−3^	(3)
*K*_*H2,AD*_	*S*_*S*_ affinity constant for H_2_ consumption	0.0018	0.0018	g H_2_ m^−3^	(4)
*K*_*I*_	*Inhibition constant on abiotic nitrate reduction*	0.0084	7.1	kg COD m^−3^	estimated
*f*_*d*_	*Fraction of biomass that is biodegradable*	0.8	0.8	–	(1)
*a*	*Constant, order for S*_*NO3*_	1.5	1	–	estimated
*b*	*Constant, order for S*_*Fe0*_	2.7	0	–	estimated
*c*	*Constant for temperature kinetics*	–	0.1	–	estimated
	Source: (1) Rittmann and McCarty^21^; (2) Xiao *et al*.^22^; (3) Kurt *et al*.^23^; (4) Smith *et al*.^16^				

**Table 2:** The new mathematical model to describe nitrate removal in NZVI-based microbial hydrogen-utilizing denitrification.

Variable Process	*S*_*H2*_	*S*_*NH4*_	*S*_*NO3*_	*S*_*N2*_	*S*_*Fe(0)*_	*S*_*Fe(II)*_	*X*_*AD*_	*X*_*I*_	Kinetic rate expressions
	g H_2_/m^3^	g N/m^3^	g N/m^3^	g N/m^3^	mol/m^3^	mol/m^3^	Kg COD/m^3^	Kg COD/m^3^	
1 abiotic nitrate reduction		1	−1		−4/14	4/14			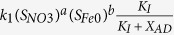
2 Fe(0) corrosion	2				−1	1			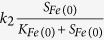
3 denitrification growth	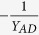		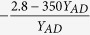	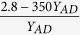			1		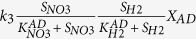
4 endogenous respiration			−1				−1		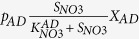
5 inactivation							−1	1-f_d_	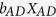

It should be noted that a temperature-dependent kinetics (k = k(25 °C)e^(c(T−25 °C))^) as described in Henze *et al*.^32^ was applied to each process 1–3 to describe the effect of temperature on nitrate removal in Case II (k is the maximum reaction rate at temperature T °C; k(25 °C) is the maximum reaction rate at temperature 25 °C, c is a constant for calibration).
